# Histone deacetylase (HDAC)-1, −2, −4 and −6 expression in human pancreatic adenocarcinoma: associations with clinicopathological parameters, tumor proliferative capacity and patients’ survival

**DOI:** 10.1186/s12876-015-0379-y

**Published:** 2015-10-26

**Authors:** Constantinos Giaginis, Christos Damaskos, Ioannis Koutsounas, Adamantia Zizi-Serbetzoglou, Nicolaos Tsoukalas, Efstratios Patsouris, Gregorios Kouraklis, Stamatios Theocharis

**Affiliations:** 1 First Department of Pathology, Medical School, National and Kapodistrian University of Athens, Athens, Greece; 2 Department of Food Science and Nutrition, School of Environment, University of the Aegean, Mitropoliti Ioakeim 2, 81400 Myrina, Limnos, Greece; 3 Second Department of Propedeutic Surgery, Medical School, University of Athens, Athens, Greece; 4 Department of Pathology, Tzaneio General Hospital, Piraeus, Greece

**Keywords:** Histone deacetylase, Pancreatic adenocarcinoma, Immunohistochemistry, Clinicopathological parameters, Patients’ survival

## Abstract

**Background:**

Histone deacetylases (HDACs) have been associated with malignant tumor development and progression in humans. HDAC inhibitors (HDACIs) are currently being explored as anti-cancer agents in clinical trials. The present study aimed to evaluate the clinical significance of HDAC-1, −2, −4 and −6 protein expression in pancreatic adenocarcinoma.

**Methods:**

HDAC-1, −2, −4 and −6 protein expression was assessed immunohistochemically on 70 pancreatic adenocarcinoma tissue specimens and was statistically analyzed with clinicopathological characteristics and patients’ survival.

**Results:**

Enhanced HDAC-1 expression was significantly associated with increased tumor proliferative capacity (*p* = 0.0238) and borderline with the absence of lymph node metastases (*p* = 0.0632). Elevated HDAC-4 expression was significantly associated with the absence of organ metastases (*p* = 0.0453) and borderline with the absence of lymph node metastases (*p* = 0.0571) and tumor proliferative capacity (*p* = 0.0576). Enhanced HDAC-6 expression was significantly associated with earlier histopathological stage (*p* = 0.0115) and borderline with smaller tumor size (*p* = 0.0864). Pancreatic adenocarcinoma patients with enhanced HDAC-1 and −6 expression showed significantly longer survival times compared to those with low expression (*p* = 0.0022 and *p* = 0.0113, respectively), while a borderline association concerning HDAC-2 expression was noted (*p* = 0.0634).

**Conclusions:**

The present study suggested that HDACs may be implicated in pancreatic malignant disease progression, being considered of clinical utility with potential use as therapeutic targets.

## Background

Acetylation of DNA-bound core histones constitutes an essential mechanism of epigenetic transcriptional regulation [1, 2]. Histone acetylation is regulated by two opposing classes of enzymes: histone acetyltransferases (HATs), which transfer the acetyl moiety from acetyl coenzyme A to specific lysine residues of histones and histone deacetylases (HDACs), which catalyze their removal in order to re-establish the positive charge in the histones [2, 3]. To date, a total of 19 HDACs have been identified in humans, being grouped into three distinct classes, with corresponding homology to the yeast S. Cerevisiae. Among them, class I HDACs is represented by HDAC-1, −2, −3, and −8, with homology to the yeast Rpd3, where the catalytic domain constitutes the majority of the protein, and class II HDACs comprises of HDAC-4, −5, −6, −7, −9, and 10 [1–3].

Both HATs and HDACs have been considered as crucial regulators of cell proliferation, differentiation and apoptosis in various hematological and solid malignancies [4, 5]. Aberrant deacetylation of histones by enhanced HDAC activity in human tumors has been shown to lead to conformational changes within nucleosome, which results in transcriptional repression of genes involved in differentiation and negative regulation of cell proliferation, migration and metastasis [6, 7]. Importantly, several HDAC inhibitors (HDACIs) have been shown to induce cell cycle growth arrest in both normal and transformed cells and activate the extrinsic and intrinsic pathways of apoptosis [6, 7]. Both *in vitro* and *in vivo* data and ongoing clinical trials have recently revealed that HDACIs could be used against different solid tumors and hematological malignancies, consisting one of the most promising classes of new anticancer agents [8–13].

Pancreatic cancer is one of the most lethal malignant tumors presenting extremely poor prognosis [14, 15]. Tumor resection is performed in 9–36 % of patients and the 5-year survival rate of patients who have undergone resection is only 19–24 % [14, 15]. Hence, there is a strong demand for novel specific markers to be explored in respect to pancreatic adenocarcinoma patients’ management and prognosis. Moreover, chemotherapy, such as treatment with 5-fluorouracil or gemcitabine, is not potentially capable of contributing to significant survival benefit according to the available literature data, although their combination is associated with a small survival advantage of about 4 to 8 weeks [16]. In this aspect, the establishment of alternative therapeutic approaches for the treatment of pancreatic cancer remains a great challenge.

In the last few years, HDACs have been shown to be overexpressed in many human malignancies, including mobile tongue, thyroid, lung, gastric, colorectal, breast, ovarian, endometrial, pancreatic, prostate, brain and renal cell carcinomas as also in hematological malignancies [17–31]. Notably, most of the above studies suggested that HDACs expression was directly associated with tumor dedifferentiation, enhanced proliferation and invasion, disease stage and patients’ prognosis [17–31]. However, the available data so far, evaluating the immunohistochemical expression of HDACs in pancreatic adenocarcinoma remain scarce, being only restricted to HDAC-1 member [24]. In view of the above considerations, the present study aimed to assess immunohistochemically the expression of HDAC-1, −2, −4 and −6 in tumoral specimens obtained from pancreatic adenocarcinoma patients. We also aimed to evaluate the association of HDAC-1, −2, −4 and −6 expression with clinicopathological characteristics, tumor proliferative capacity and patients’ survival.

## Methods

### Clinical material

Seventy pancreatic ductal adenocarcinoma specimens obtained from equal number of patients who underwent surgical resection due to pancreatic cancer were included in this study. The study was approved by the institutional ethical committee of the Medical School of the University of Athens. Informed consent to use their biological samples and clinical data for research purposes was signed by all patients under study. None of the patients received any kind of anti-cancer treatment prior to surgery. Forty-four of the patients were men (62.9 %) and 26 women (37.1 %), with a mean age of 66.77 ± 8.94 years (range 33–84 years). The cases were classified based on the World Health Organization criteria for histological grading as: well in 9 (12.9 %); moderately in 51 (72.8 %); poorly differentiated in 10 (14.3 %) [32]. Tumor staging was assessed using the 5th edition of the Tumour, Node, Metastasis (TNM) and the American Joint Committee on Cancer (AJCC) Grouping system [33, 34]. In fact, tumors were classified as: T1 in 4 (5.7 %), T2 in 8 (11.4 %), T3 in 48 (68.6 %) and T4 in 10 (14.3 %) cases. Thirty-three (47.1 %) were lymph node negative (N0), and 37 (52.9 %) were regional lymph node positive (N1). Organ metastasis was noted in 4 (5.7 %) out of 70 patients examined. According to the AJCC classification, 10 (14.3 %) cases were characterized as stage I, 46 (65.7 %) as stage II, 10 (14.3 %) as stage III and 4 (5.7 %) as stage IV. The patients were followed up until death for a time interval of 4 up to 21 months with a mean survival time of 8.69 ± 3.57 months. Overall survival was defined as the time interval between the date of surgery and the date of death due to pancreatic adenocarcinoma. At the time of the last follow-up, all patients had died from the disease.

### Immunohistochemistry

Immunostainings for HDAC-1, −2, −4 and −6 were performed on individual formalin-fixed, paraffin-embedded pancreatic adenocarcinoma tissue sections using rabbit polyclonal anti-HDAC-1 (H-51, sc-7872, Santa Cruz Biotechnology, Santa Cruz, CA, USA) and anti-HDAC-2 (H-54, sc-7899, Santa Cruz Biotechnology) IgG antibodies and mouse monoclonal anti-HDAC-4 (A-4, sc-46672, Santa Cruz Biotechnology) IgG_2b_ and anti-HDAC-6 (D-11, sc-28386, Santa Cruz Biotechnology) IgG_2a_ antibodies. Briefly, 4 μm thick tissue sections were dewaxed in xylene and were brought to water through graded alcohols. Antigen retrieval was performed by microwaving slides in 10 mM citrate buffer (pH 6.1) for 15 min (min) at high power, according to the manufacturer’s instructions. To remove the endogenous peroxidase activity, sections were then treated with freshly prepared 0.3 % hydrogen peroxide in methanol in the dark, for 30 min, at room temperature. Non-specific antibody binding was blocked using Sniper, a specific blocking reagent for mouse and rabbit primary antibodies (Sniper, Biocare Medical, Concord, California, USA) for 5 min. The sections were incubated for 1 h (h), at room temperature, with the primary antibodies against HDAC-1, −2, −4 and −6 diluted 1:200 in phosphate buffered saline (PBS) according to the manufacturer’s instructions. Sections were then incubated at room temperature with biotinylated linking reagent (Biocare Medical) for 10 min, followed by incubation with peroxidase-conjugated streptavidin label (Biocare Medical) for 10 min. The resultant immune peroxidase activity was developed using a DAB substrate kit (Vector Laboratories, California, USA) for 10 min. Sections were counterstained with Harris’ hematoxylin and mounted in Entellan (Merck, Darmstadt, Germany). Appropriate negative controls were performed by omitting the primary antibody and/or substituting it with an irrelevant anti-serum. As positive control, mobile tongue squamous and thyroid carcinoma tissue sections with known increased HDAC-1, −2, −4 and −6 expression were used [17, 18]. Isotype controls were not performed. The tumour cells’ proliferative capacity was assessed by Ki-67 immunohistochemical expression, as previously described [17, 18].

### Evaluation of immunohistochemistry

Immunohistochemical evaluation was performed by counting at least 1000 tumor cells in each case by two independent observers (S.T. and E.P.) blinded to the clinical data, with complete observer agreement. Specimens were considered “positive” for HDAC-1, −2, −4 and −6 when more than 5 % of tumor cells within the section were positively stained. HDAC-1, −2, −4 and −6 immunoreactivity was scored according to the percentage of positive tumor cells as 0: negative staining- 0–4 % of cells positive; 1: 5–24 % of cells positive; 2: 25–49 % of cells positive; 3: 50–100 % of cells positive, and its intensity as 0: negative staining, 1: mild staining; 2: intermediate staining; 3: intense staining. Finally, the expression of HDAC-1, −2, −4 and −6 was classified as low; if the total score was 0 or 2 and high; if the total score was ≥3 [17, 18]. In this way, we ensure that each group has a sufficient and more homogeneous number of cases in order to be comparable with the other groups [17, 18]. Ki-67 immunoreactivity was classified according to the percentage of positively stained tumor nuclei exceeding the mean percentage value into two categories (below and over median value), as previously reported [17, 18].

### Statistical analysis

Chi-square test was used to assess the associations of HDAC-1, −2, −4 and −6 proteins’ expression with clinicopathological variables. Survival curves were constructed using the Kaplan-Meier method and the differences between the curves were compared by the log rank test. A Cox proportional-hazard regression model was developed to evaluate the association between the potential prognostic marker and overall survival, at multivariate level. A p-value less than 0.05 was considered the limit of statistical significance. SPSS for Windows Software was used for all analyses (SPSS Inc., 2003, Chicago, USA).

## Results

### Clinical significance of HDAC-1 expression in pancreatic adenocarcinoma

**Fig. 1:**
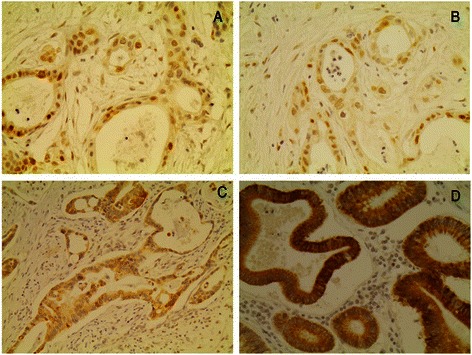
Representative immunostainings of pancreatic adenocarcinoma tumor cells presenting high expression of **a**. HDAC-1, **b**. HDAC-2, **c**. HDAC-4 and **d**. HDAC-6. Streptavidin-biotin-peroxidase, DAB chromogen, Harris hematoxylin counterstain (original magnification X200)

Forty-five (64.3 %) out of 70 pancreatic adenocarcinoma cases were found HDAC-1 positive. The pattern of HDAC-1 distribution was nuclear in all positive cases (Fig. 1a). High HDAC-1 expression was noted in 28 (40.0 %) out of 70 pancreatic adenocarcinoma cases. Non-neoplastic sites of pancreatic tissues were found negative for HDAC-1 (data not shown).

In crosstabulation, high HDAC-1 expression was significantly associated with increased tumor proliferative capacity (Table 1, *p* = 0.0238). High HDAC-1 expression was borderline more frequently observed in pancreatic adenocarcinoma patients with absence of lymph node metastases (Table 1, *p* = 0.0632). High HDAC-1 expression was also more frequently observed in pancreatic adenocarcinoma patients presenting smaller tumor size and earlier histopathological stage, at a non significant level though (Table 1, *p* = 0.1544 and *p* = 0.1127, respectively).

**Table 1:** Associations of HDAC-1 and −2 expression with clinicopathological parameters in 70 pancreatic adenocarcinoma patients

Clinicopathological characteristics	HDAC-1 expression			HDAC-2 expression		
	Low (%)	High (%)	*p*-value	Low (%)	High (%)	*p*-value
*N* = 70	42 (60.0)	28 (40.0)		35 (50.0)	35 (50.0)	
Age (mean ± SD;ys)			0.4880			0.8082
≤66.77 ± 8.94 yrs	16 (22.9)	13 (18.6)		14 (20.0)	15 (21.4)	
>66.77 ± 8.94 yrs	26 (37.1)	15 (21.4)		21 (30.0)	20 (28.6)	
Gender			0.4191			0.6207
Male	28 (40.0)	16 (22.9)		23 (32.9)	21 (30.0)	
Female	14 (20.0)	12 (17.1)		12 (17.1)	14 (20.0)	
Histopathological grade			1.0000			0.4945
I + II	36 (51.4)	24 (34.3)		29 (41.4)	31 (44.3)	
I II	6 (8.6)	4 (5.7)		6 (8.6)	4 (5.7)	
pT			0.1544			0.2046
T1 + T2	5 (7.1)	7 (10.0)		4 (5.7)	8 (11.4)	
T3 + T4	37 (52.9)	21 (30.0)		31 (44.3)	27 (38.6)	
pN			0.0632			0.2312
0	16 (22.9)	17 (24.3)		19 (27.1)	14 (20.0)	
1	26 (37.1)	11 (15.7)		16 (22.9)	21 (30.0)	
pM			0.5282			1.0000
0	39 (55.7)	27 (38.6)		33 (47.1)	33 (47.1)	
1	3 (4.3)	1 (1.4)		2 (2.9)	2 (2.9)	
pStage			0.1127			0.2320
I + II	31 (44.3)	25 (35.7)		26 (37.1)	30 (42.9)	
III + IV	11 (15.7)	3 (4.3)		9 (12.9)	5 (7.1)	
Ki-protein statement			0.0238			0.4703
≤ median value	28 (40.0)	11 (15.7)		21 (30.0)	18 (15.7)	
> median value	14 (20.0)	17 (24.3)		14 (20.0)	17 (24.3)	

**Fig. 2:**
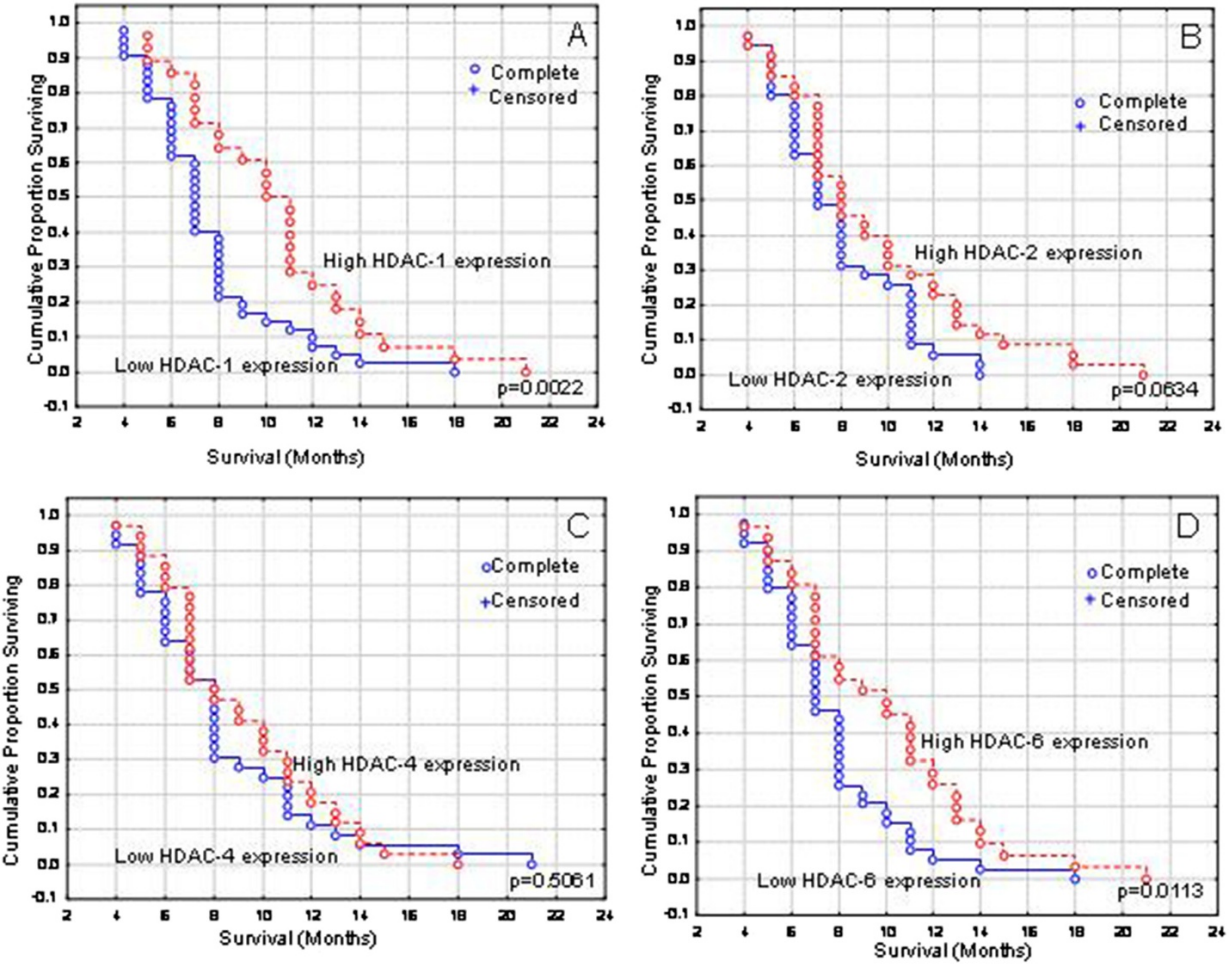
Kaplan-Meier survival analysis stratified according to **a**. HDAC-1, **b**. HDAC-2, **c**. HDAC-4 and **d**. HDAC-6 expression in pancreatic adenocarcinoma patients

Kaplan-Meier survival curves indicated that pancreatic adenocarcinoma patients presenting high HDAC-1 expression had significantly longer survival times compared to those with low expression (Fig. 2a, Table 2, log-rank test, *p* = 0.0022). In multivariate analysis, histopathological stage but not HDAC-1 expression was identified as independent prognostic factor of patients’ survival (Table 3, Cox-regression analysis, *p* < 0.001and *p* = 0.0521, respectively).

**Table 2:** Association of clinicopathological parameters and HDAC-1, −2, −4 and −6 expression with patients’ survival: Univariate analysis

Clinicopathological variables		Median overall survival (95 % CI)	*p*-value
Age	<66.77 yrs	8.0 (6.7–9.3)	0.3876
	≥66.77 yrs	8.0 (7.2–8.8)	
Gender	Male	8.0 (7.3–8.7)	0.1343
	Female	7.0 (6.2–7.8)	
Histopathological grade	I + II	8.0 (7.2–8.8)	0.8963
	III	7.0 (5.8–8.2)	
pT	T1 + T2	11.0 (5.3–16.7)	<0.0001
	T3 + T4	7.0 (6.2–7.8)	
pN	N0	11.0 (9.8–12.2)	<0.0001
	N1	7.0 (6.5–7.5)	
pM	M0	8.0 (7.2–8.8)	<0.0001
	M1	4.0 (3.9–5.1)	
pStage	I + II	8.0 (6.9–9.2)	<0.0001
	III + IV	5.0 (3.9–6.0)	
Ki-67 protein statement	≤ median	8.0 (7.1–8.7)	0.8581
	> median	7.0 (5.9–8.1)	
HDAC-1 expression	Low	7.0 (6.3–7.7)	0.0022
	High	10.0 (8.8–11.2)	
HDAC-2 expression	Low	7.0 (5.9–8.1)	0.0634
	High	8.0 (6.1–9.9)	
HDAC-4 expression	Low	8.0 (7.1–8.9)	0.5061
	High	8.0 (6.7–9.3)	
HDAC-6 expression	Low	7.0 (6.2–7.8)	0.0113
	High	10.0 (7.6–12.4)

**Table 3:** Multivariate analysis for histopathological stage and HDAC-1 expression

Clinicopathological variables	Overall survival	
	HR (95 % CI)	*p*-value
Histopathological stage (I + II / III + IV)	0.290 (0.151–0.559)	<0.0001
HDAC-1 expression (Low/ ≥ High)	1.672 (0.995–2.809)	0.0521

### Clinical significance of HDAC-2 expression in pancreatic adenocarcinoma

Forty-nine (74.2 %) out of 70 pancreatic adenocarcinoma cases were found HDAC-2 positive. The pattern of HDAC-2 distribution was nuclear in all positive cases (Fig. 1b). High HDAC-2 expression was noted in 35 (50.0 %) out of 70 pancreatic adenocarcinoma cases. Non-neoplastic sites of pancreatic tissues were found negative for HDAC-2 (data not shown).

In crosstabulation, HDAC-2 expression was not significantly associated with any clinicopathological parameters examined (Table 1). High HDAC-2 expression was more frequently observed in pancreatic adenocarcinoma patients presenting smaller tumor size, absence of lymph node metastases and earlier histopathological stage, at a non significant level though (Table 1, *p* = 0.2046, *p* = 0.2312 and *p* = 0.2320, respectively). Kaplan-Meier survival curves indicated that pancreatic adenocarcinoma patients presenting high HDAC-2 expression had marginally longer survival times compared to those with low expression (Fig. 2b, Table 2, log-rank test, *p* = 0.0634).

Concomitant HDAC-1/HDAC-2 expression was not significantly associated with any clinicopathological parameters (*p* > 0.05, data not shown). Kaplan-Meier survival curves indicated that pancreatic adenocarcinoma patients presenting concomitant high HDAC-1/HDAC-2 expression had significantly longer survival times compared to those with low expression (log-rank test, *p* = 0.0255). Concomitant HDAC-1/HDAC-2 expression was not remained significant in multivariate analysis (*p* > 0.05, data not shown).

### Clinical significance of HDAC-4 expression in pancreatic adenocarcinoma

Forty-seven (69.1 %) out of 70 pancreatic adenocarcinoma cases were found HDAC-2 positive. The pattern of HDAC-4 distribution was cytoplasmic in all positive cases (Fig. 1c). High HDAC-4 expression was noted in 34 (48.6 %) out of 70 pancreatic adenocarcinoma cases. Non-neoplastic sites of pancreatic tissues were found negative for HDAC-4 (data not shown).

In crosstabulation, high HDAC-4 expression was significantly associated with the absence of organ metastases (Table 4, *p* = 0.0453). High HDAC-4 expression was more frequently observed in pancreatic adenocarcinoma patients with absence of lymph node metastases and increased tumor proliferative capacity, at a non significant level though (Table 4, *p* = 0.0571 and *p* = 0.0576, respectively). High HDAC-4 expression was also more frequently observed in pancreatic adenocarcinoma patients presenting earlier histopathological stage, at a non significant level though (Table 4, *p* = 0.2818). HDAC-4 expression was not associated with patients’ survival (Fig. 2c, Table 2, log-rank test, *p* = 0.5061).

**Table 4:** Associations of HDAC-4 and −6 expression with clinicopathological parameters in 70 pancreatic adenocarcinoma patients

Clinicopathological characteristics	HDAC-4 expression			HDAC-6 expression		
	Low (%)	High (%)	*p*-value	Low (%)	High (%)	*p*-value
*N* = 70	36 (51.4)	34 (48.6)		39 (55.7)	31 (44.3)	
Age (mean ± SD;ys)			0.9668			0.2920
≤66.77 ± 8.94 yrs	15 (21.4)	14 (20.0)		14 (20.0)	15 (21.4)	
>66.77 ± 8.94 yrs	21 (30.0)	20 (28.6)		25 (35.7)	16 (22.9)	
Gender			0.7557			0.2157
Male	22 (31.4)	22 (31.4)		27 (38.6)	17 (24.3)	
Female	14 (20.0)	12 (17.1)		12 (17.1)	14 (20.0)	
Histopathological grade			0.4347			0.7682
I + II	32 (45.7)	28 (40)		33 (47.1)	27 (38.6)	
III	4 (5.7)	6 (8.6)		6 (8.6)	4 (5.7)	
pT			0.4573			0.0864
T1 + T2	5 (7.1)	7 (10.0)		4 (5.7)	8 (11.4)	
T3 + T4	31 (44.3)	27 (38.6)		35 (50.0)	23 (32.9)	
pN			0.0571			0.1026
0	13 (18.6)	20 (28.6)		15 (21.4)	18 (25.7)	
1	23 (32.9)	14 (20.0)		24 (34.3)	13 (18.6)	
pM			0.0453			0.0663
0	32 (45.7)	34 (48.6)		35 (50.0)	31 (44.3)	
1	4 (5.7)	0 (0.0)		4 (5.7)	0 (0.0)	
pStage			0.2818			0.0115
I + II	27 (38.6)	29 (41.4)		27 (38.6)	29 (41.4)	
III + IV	9 (12.9)	5 (7.1)		12 (17.1)	2 (2.9)	
Ki-protein statement			0.0576			0.5379
≤ median value	24 (34.3)	15 (21.4)		23 (32.9)	16 (22.9)	
> median value	12 (17.1)	19 (27.1)		16 (22.9)	15 (21.4)	

### Clinical significance of HDAC-6 expression in pancreatic adenocarcinoma

Forty-one (63.1 %) out of 70 pancreatic adenocarcinoma cases were found HDAC-6 positive. The pattern of HDAC-6 distribution was cytoplasmic in all positive cases (Fig. 1d). High HDAC-6 expression was noted in 31 (44.3 %) out of 70 pancreatic adenocarcinoma cases. Non-neoplastic sites of pancreatic tissues were found negative for HDAC-6 (data not shown).

In crosstabulation, HDAC-6 expression was significantly associated with earlier histopathological stage (Table 2, *p* = 0.0115). High HDAC-6 expression was more frequently observed in pancreatic adenocarcinoma patients with smaller tumor size and absence of organ metastases, at a non significant level though (Table 4, *p* = 0.0864 and *p* = 0.0663, respectively). High HDAC-6 expression was more frequently observed in younger and female pancreatic adenocarcinoma patients, as well as in those with absence of lymph node metastases, at a non significant level though (Table 4, *p* = 0.2920, *p* = 0.2157 and *p* = 0.1026, respectively).

Kaplan-Meier survival curves indicated that pancreatic adenocarcinoma patients presenting high HDAC-6 expression had significantly longer survival times compared to those with low expression (Fig. 2d, Table 2, log-rank test, *p* = 0.0113). In multivariate analysis, histopathological stage but not HDAC-6 expression was identified as independent prognostic factor of patients’ survival (Table 5, Cox-regression analysis, *p* < 0.001and *p* = 0.2193, respectively).

**Table 5:** Multivariate analysis for histopathological stage and HDAC-6 expression

Clinicopathological variables	Overall survival	
	HR (95 % CI)	*p*-value
Histopathological stage (I + II / III + IV)	0.282 (0.143–0.556)	<0.0001
HDAC-6 expression (Low/ ≥ High)	0.716 (0.421–1.220)	0.2193

Concomitant HDAC-1/HDAC-2/HDAC-4/HDAC-6 expression was not associated with either any clinicopathological parameter or patients’ prognosis (*p* > 0.05, data not shown).

## Discussion

In the last few years, HDACs have been considered as crucial regulators of cell proliferation, differentiation and apoptosis in various hematological and solid malignancies [6–13]. HDACs overexpression has been described in several types of human malignancy, being associated with crucial clinicopathological parameters for patients’ management and prognosis [17–31]. However, the assessment of their clinical significance in pancreatic adenocarcinoma remains scarce.

In view of above considerations, the present study assessed for the first time the clinical significance of HDAC-1, −2, −4 and −6 expression in pancreatic adenocarcinoma. We found that approximately half of the examined cases presented high HDAC-1, −2, −4 and −6 expression (40.0 %, 50.0 %, 48.6 % and 44.3 %, respectively). Moreover, all the examined cases showed negative HDAC-1, −2, −4 and −6 immunostaining in non-neoplastic sites of pancreatic tissues. The elevated expression levels of HDAC-1, −2, −4 and −6 in pancreatic adenocarcinoma may reinforce the therapeutic utility of HDAC targeting in pancreatic cancer chemoprevention, taking into consideration the anti-cancer properties of HDACIs in cell proliferation, differentiation and apoptosis [6–13]. In this context, the cdk inhibitor p21 was shown to be of the most commonly induced genes in various pancreatic cancer cell lines by several HDACIs [6, 8]. Transcriptional induction of p21 was associated with G1 cell cycle arrest and growth inhibition. HDACIs also resulted in cell cycle arrest and growth inhibition by transcriptional activation of other cell cycle regulatory genes such as p16, p27, cyclin E and gelsolin, while inhibition of cyclins A, B1, D1 and D2 were also noted in many cancer cell lines. Another mechanism of action for HDACIs concerns the up-regulation of pro-apoptotic proteins Bax, Bad and Bim, and the down-regulation of the anti-apoptotic ones such as Bcl-2, Bcl-XL and surviving [6–8]. HDACIs also reduced the expression of angiogenetic factors, such as VEGF receptors −1 and −2, and affected the expression of a panel of metastasis promoting genes [6, 8]. Additionally, HDACIs exhibited antiproliferative effects in cancer animal models. Various pancreatic carcinoma cell lines were grown as xenografts in nude mice and treated with HDACIs [6–8]. For most cell lines used, anti-tumor activity and dose-dependent response were observed, with reduction of cell proliferation and induction of apoptosis. Notably, the reduction of pancreatic tumor weight was achieved with minimal toxicity [6–8].

In the present study, HDAC-1 and −2 subcellular distribution was nuclear, whereas HDAC-4 and −6 distribution was cytoplasmic. In accordance to the above findings, class I HDACs are mostly localized within the nucleus due to the lack of a nuclear export signal, whereas class II HDACs shuttle between nucleus and cytoplasm in response to certain cellular signals [2, 3]. In this aspect, knockout analysis of different class I and class II HDAC proteins indicated that class I HDACs play a role in cell survival and proliferation, whereas class II HDACs may have tissue-specific roles [2, 3].

The present study further showed that HDAC-1, −2, −4 and −6 immunohistochemical expression was associated with clinicopathological characteristics, which are considered crucial for patients’ management and prognosis. Notably, elevated HDAC-1 expression was significantly associated with increased tumor proliferative capacity, enhanced HDAC-4 expression with the absence of distant metastases and elevated HDAC-6 expression with earlier histopathological stage. Trends of correlation with lymph node metastases and tumor size were also obtained. Enhanced HDAC-1 and −6 expression was further associated with better overall patients’ survival. In agreement with the present findings, recent studies supported evidence that HDACs expression was directly associated with tumor differentiation, proliferation and invasion, disease stage and patients’ prognosis in several human malignancies [6, 8, 35]. According to a comprehensive and critical review by Weichert, the majority of the existing studies reported an enhanced expression of class I HDAC isoforms in solid human tumours, both on mRNA and protein level [35]. In most studies, class I HDAC expression was increased in locally advanced, dedifferentiated and strongly proliferating tumours [35]. In some but not all tumour entities elevated class I HDAC expression was associated with poor patient survival [35]. However, an association of elevated class I HDAC expression with improved patients’ prognosis for selected tumour types has also been reported [35]. In contrast to class I isoforms, expression of class II HDACs has been found reduced in tumours and high expression of these isoforms in some tumour types predicted better patients’ outcome [35]. In fact and in accordance with the present study, elevated HDAC-1 expression was associated with absence of axillary lymph node involvement, smaller tumor size, well tumor differentiation and better disease-free and overall survival in breast cancer [28]. Moreover, another study documented that enhanced HDAC-6 expression was associated with smaller tumor size and better patients’ survival in invasive breast cancer [36].

Concerning pancreatic adenocarcinoma, only one study conducted on 55 cases has currently evaluated the clinical significance of HDAC-1 member [24]. In this study, enhanced HDAC-1 expression was significantly associated with tumor high histological grade and increased proliferative capacity, as also with advanced TNM stage and shorter patients’ survival [24]. Although inverse correlations were reported by Wang et al. [25] and us, both studies support evidence for the clinical utility of HDAC-1 expression in pancreatic adenocarcinoma. The discrepancies in the results between the studies could be ascribed to the lower number of pancreatic adenocarcinoma cases (*N* = 55) used in the above study compared to our present work (*N* = 70), as also to the different primary antibody used to detect HDAC-1 immunoreactivity and the different demographics. Moreover, in the above study the evaluation criteria to semi-quantify HDAC-1 expression was based on the percentage of positively stained tumor cells, whereas in our work they were based on both the percentage of positively stained tumor cells and the staining intensity.

Recently, HDACIs have been introduced into clinical trials, especially in pre-treated and multiply relapsed patients at an advanced cancer stage [5–11]. The first HDACIs tested in clinical trials have shown encouraging anti-tumor effects, at dosages well-tolerated by patients. Importantly, vorinostat was the first HDACI to be approved for clinical use in treating patients with hematological malignancy (cutaneous T-cell lymphoma) [37]. HDACIs alone and in combination with a variety of cytotoxic or other targeted anticancer agents are currently being tested. Currently, at least ten different HDACIs are under phase II or III clinical studies for the treatment of hematological and solid tumors [5–11]. However, clinical data for HDACIs on patients with pancreatic cancer remains inadequate and only a few studies have included patients suffering from this type of neoplasm [6, 8]. Additionally, the number of pancreatic cancer patients that entered HDACIs phase II/III trials, among others with advanced solid tumors, is very limited. Although HDACIs are recognized as one of the most promising agents, more studies recruiting for candidates suffering from pancreatic cancer remain to determine the efficiency of these therapies [6, 8].

## Conclusion

The present study for the first time showed that elevated HDACs expression were associated with favourable clinicopathological parameters, such as earlier histopathological stage, smaller tumor size and absence of lymph node and organ metastases, which are considered crucial for pancreatic carcinoma patients’ management and prognosis. Of even more clinical significance are the data, supporting the association of HDAC-1 and −6 expression with patients’ survival. These findings provided evidence for a potential important role of HDACs in the biological mechanisms governing pancreatic malignant disease progression. The potential implication of HDACs in the pancreatic adenocarcinoma, along with a few observations in this field, point out the necessity for further studies in order to clarify the potential role of these molecules and their possible use in the established therapeutic regimens of this type of malignancy.

## Abbreviations

HATs: Histone acetyltransferases
AJCC: American joint committee on cancer
HDACs: Histone deacetylases
HDACIs: Histone deacetylases inhibitors
TNM: Tumour, node, metastasis
VEGF: Vascular endothelial growth factor
